# Role of C-reactive Protein as an Indicator for Determining the Outcome of Sepsis

**DOI:** 10.5005/jp-journals-10071-23105

**Published:** 2019-01

**Authors:** Meeval M Anush, Vijay K Ashok, Ramakrishna IN Sarma, Sreehari K Pillai

**Affiliations:** 1 Kerala Institute of Medical Sciences Hospital, Trivandrum, Kerala, India; 2 Department of Internal Medicine, MES Medical College, Perinthalmanna, Kerala, India; 3 Department of Internal Medicine, Pushpagiri Medical College, Thiruvalla, Kerala, India; 4 NMC Speciality Hospital, Abu Dhabi, United Arab Emirates

**Keywords:** C-reactive protein, Sepsis, Sequential organ failure assessment score

## Abstract

**Background and aims:**

It has been observed that after any injury which is acute and also in the setting of inflammation or infection, the synthesis and secretion of C-reactive protein (CRP) rises within a few hours. The current study monitors CRP in patients presenting with sepsis and attempts to prove that it is one of the most reliable tests in determining the resolution and predicting the outcome.

**Materials and methods:**

During 12 months, 97 individuals with culture-proven sepsis were included, and a prospective observational study was done. Patients were assessed clinically by recording vitals, mean arterial pressure, Glasgow coma scale score, sequential organ failure assessment (SOFA) score as well as assessment of arterial blood gas and other blood investigations, which included CRP, total white cell count, differential count, serum creatinine, serum bilirubin on day 0, day 2 and day 5 after initiating antibiotics. To test the statistical significance of the difference in mean percentage changes of the different study variables between living and expired groups at day 2 and day 5, Wilcoxon's rank sum test was applied due to the non-normal distribution of values and small sample sizes.

**Results:**

The percentage drop of the mean of CRP from day 0 to day 2 was 23.33% in the living group, and there was an increase of 4.73 % in the expired group. The percentage drop of the mean of CRP on day 5 when compared to day 0, was significant in the living group.

**Conclusion:**

C-reactive protein (CRP) is a more useful tool in predicting improvement and outcome in patients admitted with sepsis when compared to scoring systems like SOFA score.

**Abbreviations:**

AIMS: Amrita Institute of Medical Sciences, C1q: Complement 1q, CRP: C-reactive Protein, PCT: Procalcitonin, SOFA: Sequential organ failure assessment

**How to cite this article:**

Anush MM, Ashok VK, Sarma RIN, Pillai SK. Role of C-reactive Protein as an Indicator for Determining the Outcome of Sepsis. Indian Journal of Critical Care Medicine, January 2019; 23(1):11-14.

## INTRODUCTION

Acute inflammatory conditions, as well as infections, make the liver produce CRP, which is a protein belonging to a family of proteins called pentraxins, having a key role in activation of the complement system by means of the C1q complex, thereby setting into action one of our major defense mechanisms.

A CRP is an acute phase reactant and a sensitive marker when an individual has sepsis. When there is an acute infection or inflammation, the concentration of CRP in the blood can be measured, which can be elevated as early as two hours after the triggering event, reaching peak values in 48 hours.

Even though the liver synthesizes CRP in response to certain factors released by macrophages and adipocytes, owing to its comparatively short half-life of 18 hours, CRP level in blood can be monitored frequently and utilized as a marker for resolution/worsening of infection as well as inflammation. Values can shoot up to 300 mg/L within 2 days. In a normal individual, values are generally below 3 mg/L.

Based on a research article by Coelho et al.,^1^ the prognosis is poor, if the CRP level is 0.5 times elevated from the baseline level by Day 2 (91% sensitivity, 59% specificity). Conversely, he observed that when CRP values reduced by 0.31 or more on Day 2, when compared to the value on the previous day, after starting antibiotics (day 0), the prognosis was good (75% sensitivity, 85% specificity).

Sequential organ failure assessment (SOFA) score is a scoring system which is used to predict the outcome of sepsis. The six organ systems which are assessed while using the SOFA score are the nervous system, the cardiovascular system, the hematological system, the renal system, the gastrointestinal system, and the pulmonary system. If a person is normal, the score is 0 and if he/she is on the other end of the spectrum, the score is 4, which indicates a severe abnormality. It was intended to provide the simplest daily description of organ dysfunction for use in clinical trials. It has the merit of including supportive therapy, and although increasing scores can be shown to be associated with increasing mortality it was not designed for estimation of outcome probability. This simple method has become a popular method by which to track and describe changes in mortality.

**Graph 1:**
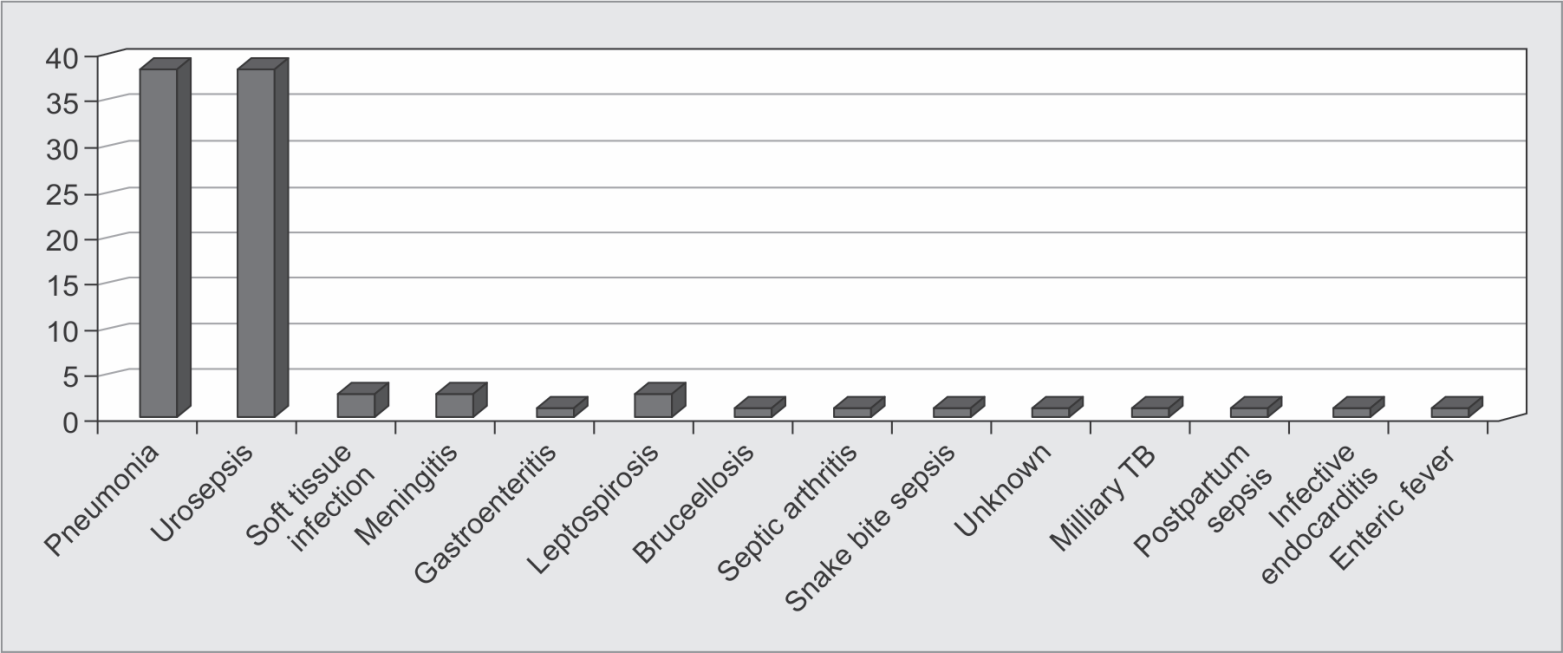
Causes of sepsis

The current study is to monitor CRP in patients presenting with sepsis and to prove that it is one of the most reliable tests in determining the resolution and predicting the outcome in comparison to other markers of sepsis-like the respiratory rate (RR), white blood cell (WBC) count and the SOFA score.

## MATERIALS AND METHODS

### Patients

During 12 months, 97 individuals found to have culture-proven sepsis were included in the study and a prospective, observational study was done. They were assessed clinically by recording vital signs, SOFA score, arterial blood gas, mean arterial pressure, Glasgow coma scale, and blood investigations, which included, CRP, total WBC count, differential count, serum creatinine, serum bilirubin on day 0, day 2 and day 5 after initiating antibiotics.

### Statistical Analysis

To test the statistical significance of the difference in mean percentage changes of the different study variables between living and expired groups at the second and fifth day, Wilcoxon's rank sum test was applied due to the non-normal distribution of values and small sample sizes.

## RESULTS

Ninety-seven patients with culture-proven sepsis were selected and serial monitoring of CRP and other parameters were studied. Out of 97 patients, 29 patients expired and 68 patients were in the living group. Patients with different causes of sepsis were taken into the study as shown in Graph 1.

**Graph 2:**
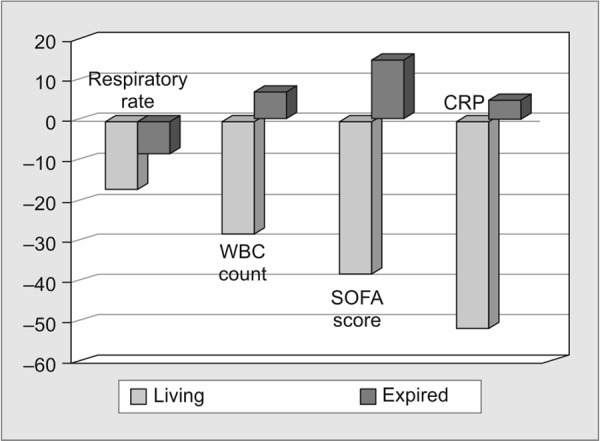
Comparison of mean percentage change of different study variables between living and expired groups on day 2

Comparison of the mean percentage change of the different study variables between the living and expired groups at day 2 was analyzed as shown in Table 1. It proved that while all the variables in case of the living group showed an increase in the percentage change, in the expired group this percentage drop was comparatively less. The differences in the mean percentage change between living and expired groups were found to be statistically significant at various levels of significance. When individuals were grouped into those who survived and those who expired, there was a commendable drop in CRP in the former group when compared to the latter group, as shown in Graph 2.

**Graph 3:**
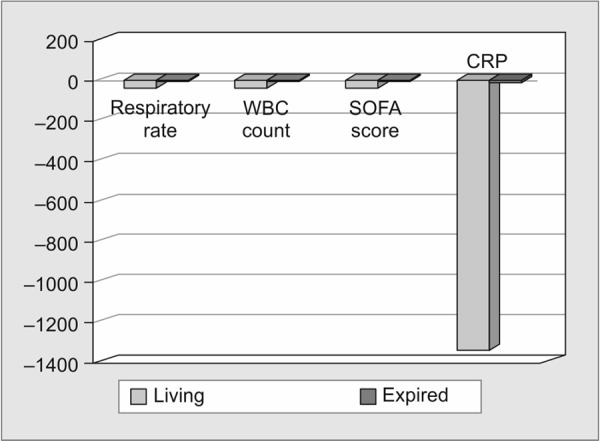
Comparison of mean percentage change of different study variables between living and expired groups on day 5

Comparison of the mean percentage change of the different study variables between the living and expired groups on the 5th day analyzed showed a decrease in percentage change by the fifth day. In the expired group, except for respiratory rate and CRP, the other variables showed an increase in mean percentage, as shown in Table 2. The decrease in the mean percentage of both these variables was very small compared to the living group. The difference in the mean percentage of CRP was not statistically significant because of the high variation in CRP values in the living group as shown in Graph 3.

**Table 1:** Comparison of mean percentage change of different study variables between living and expired groups on day 2

	*Living*	*Expired*					
*Variables*	*n*	*Mean *	*SD*	*N*	*Mean*	*SD*	*p value*
Respiratory rate	68	-17.50	16.18	29	-8.47	14.60	0.011
WBC count	68	-28.59	41.90	29	7.29	36.66	<0.001
SOFA score	68	-38.99	60.87	29	15.32	34.00	<0.001
CRP	68	52.52	95.08	29	5.30	49.11	0.003

**Table 2:** Comparison of mean percentage change of different study variables between living and expired groups on day 5

	*Living*		*Expired*				
*Variables*	*n*	*Mean*	*SD*	*n*	*Mean*	*SD*	*p-value*
Respiratory rate	68	-35.15	27.24	29	-10.65	18.51	<0.001
WBC count	68	-46.21	73.40	29	4.89	54.09	0.001
SOFA score	68	-59.27	97.79	29	12.33	50.39	<0.001
CRP	68	-1350.75	8752.00	29	-22.72	69.61	0.417

The percentage drop of mean, from day 0 to day 2 of CRP, was 23.33% in the living group and there was an increase of 4.73 % in the expired group as shown in Table 3. The percentage drop of mean, from day 0 to day 5 of CRP, was significant in the living group; a drop of 55.55%, which was higher compared to other variables (respiratory rate of 14.12%, WBC of 27.75% and SOFA score of 49.08%).

## DISCUSSION

In this study, we assessed the serum CRP values on day 0, day 2 and day 5 after initiation of antibiotics. It was observed that among those who survived, CRP showed a significantly noticeable reduction when compared to those individuals who expired.

The importance of serial monitoring of CRP in a person with sepsis lies in the fact that progressive reduction in values of serum CRP is an early indicator that sepsis is resolving and so, the serial values of CRP can aid the clinician to decide whether to modify the treatment or not, as, more often than not, progressive reduction of CRP values and clinical improvement go hand in hand.^2-4^

Even though CRP may not be specific for sepsis, when we compare it to SOFA score as well as other parameters like respiratory rate and WBC count, the response was best seen with CRP in the survivors by day 2. But percentage drop from day 0 to day 5 was statistically not significant as the decrement was very dramatic. This is probably because of the sudden decrease in CRP after the initiation of antibiotics proving a very good response to treatment.^5^

**Table 3:** Percentage drop of mean from day 0 to day 2 and day 0 to day 5 among the study variables

*Indicator*	*Mean*		*Percentage drop of mean from day 0 to day 2*		*Percentage drop of mean from day 0 to day 5*	
	*Expired*	*Living*				
RR 0	27.24	25.15	Expired	6.98 (drop)	Expired	6.98 (drop)
RR 2	25.34	21.6				
RR 5	25.34	21.6	Living	14.12 (drop)	Living	14.12 (drop)
WBC 0	12395.86	15734.41	Expired	19.17 (increase)	Expired	37.87 (increase)
WBC 2	14771.72	12565.29				
WBC 5	17090.34	11367.94	Living	20.14 (drop)	Living	27.75 (drop)
SOFA 0	5.9	3.26	Expired	13.90 (increase)	Expired	21.02 (increase)
SOFA 2	6.72	2.51				
SOFA 5	7.14	1.66	Living	23.01 (drop)	Living	49.08 (drop)
CRP 0	131.117	147.917	Expired	4.73 (increase)	Expired	11.78 (drop)
CRP 2	137.316	113.41				
CRP 5	115.669	66.073	Living	23.33 (drop)	Living	55.33 (drop)

In bacterial infections, the more specific marker is Procalcitonin (PCT), when compared to CRP. However, considering the cost factor, longer half-life and the statistically proven data of reliability of serial monitoring of CRP in resolution or worsening of sepsis, it can be adopted as "must do" investigation for patients admitted with sepsis and initiated on antibiotics.^6-9^

Elevated CRP levels along with culture positivity were found to have an association which was more statistically significant than the levels of PCT, as per a study was done in Korea.^10^

If there is worsening of CRP, measures can be taken either to rethink on the antibiotics initiated or carry out some intervention to treat the sepsis; for example, incision and drainage for an abscess.

It was observed that individuals with higher SOFA scores had poorer outcomes, and this was also an association which had statistical significance. Even though initial survival rates were comparable between those who survived and those who succumbed, it was seen that as the duration of hospitalization increased, the SOFA score worsened in the population who expired.^11^

According to the data published by Seymour et al*., *sepsis was identified in a group of patients on intensive care with suspicion for infection, when there was an increase in SOFA score by 2 or more points.^12^

In patients with sepsis, CRP is a more useful tool in predicting improvement and outcome when compared to scoring systems like SOFA score.

## CONCLUSION

Serial monitoring of CRP especially during the first 5 days of initiation of antibiotics in patients with sepsis helps in ascertaining the prognosis and predicting the possible outcome by even the third day of hospitalization. It is even possible to recognize whether the trend of the illness is improving or worsening and to assess the extent of improvement on an individual basis based on the absolute value of CRP. From the available data, it can be concluded that serially monitoring of CRP after initiating antibiotics should be the norm so that duration of antibiotic therapy can be cut short once CRP normalizes and also, patients not responding to treatment can be picked up early.
